# 10 °C Storage for the Preservation of Pig Kidney Grafts: A Porcine Autotransplant Model

**DOI:** 10.1097/TXD.0000000000001993

**Published:** 2026-08-21

**Authors:** Francisco Calderon Novoa, Kenji Nakamura, Kun Wang, Sujani Ganesh, Kaitlin Lees, Qing Rui Qu, Emma Mizdrak, Catherine Parmentier, Christian Hobeika, Samrat Ray, Nicola Sariye Pollman, Rohan John, Trevor Reichman, Lisa Robinson, Markus Selzner

**Affiliations:** 1 Ajmera Transplant Centre, Toronto General Hospital, Toronto, ON, Canada.; 2 Division of General Surgery, Department of Transplantation, University Health Network, Toronto, ON, Canada.; 3 Division of Nephrology, The Hospital for Sick Children, Toronto, ON, Canada.; 4 Toronto General Hospital Research Institute, University Health Network, Toronto, ON, Canada.; 5 Temerty Faculty of Medicine, University of Toronto, Toronto, ON, Canada.; 6 Graduate Department of Pharmaceutical Sciences, Leslie Dan Faculty of Pharmacy, University of Toronto, Toronto, ON, Canada.; 7 Royal College of Surgeons Ireland (RCSI), University of Medicine and Health Sciences, Dublin, Ireland.; 8 Department of HPB Surgery and Liver Transplantation, Beaujon Hospital, APHP, Clichy, Paris-Cité University, Paris, France.; 9 Department of General, Visceral and Vascular Surgery, Jena University Hospital, Jena, Germany.; 10 Department of Laboratory Medicine and Pathobiology, University of Toronto, Toronto, ON, Canada.; 11 Department of Pathology, University Health Network, Toronto, ON, Canada.; 12 Program in Cell Biology, The Hospital for Sick Children Research Institute, Toronto, ON, Canada.

## Abstract

**Background.:**

Optimization of organ storage is essential to improve outcomes in kidney transplantation. Recent studies in lung transplantation highlight the impact of temperature on organ preservation, leading to a shift from traditional 4 °C “on-ice” storage toward 10 °C. We sought to determine whether these findings extend to kidney transplantation using 2 distinct porcine autotransplant models.

**Methods.:**

The initial donation after circulatory death model consisted of 30 min of warm ischemia followed by a brief 7.5 h storage on-ice (n = 5) or at 10 °C (n = 5). Grafts were retransplanted, the contralateral kidney was removed, and participants were followed up for 3 d. A second model simulating donation after brain death compared 24-h storage at 10 °C (n = 3) to a contemporaneous standard ice storage cohort (n = 3), followed by transplant, contralateral nephrectomy, and 7-d follow-up.

**Results.:**

The first model revealed discrete differences in creatinine, blood urea nitrogen, sodium, potassium, urine output, creatinine clearance, and fractional sodium excretion favoring 10 °C (*P* > 0.05). Histology revealed reduced tubular injury (*P* = 0.03) and interstitial inflammation (*P* < 0.05) in the 10 °C group. Urinary neutrophil gelatin-associated lipocalin and thiobarbituric acid reactive substances were also reduced at 10 °C in the postoperative period (*P* > 0.05). However, prolonged 24-h storage at 10 °C did not benefit kidney function, with significantly worse creatinine and blood urea nitrogen values over time (*P* < 0.05).

**Conclusions.:**

The results of this initial study suggest that 10 °C storage may offer a modest protective effect in a short-storage porcine donation after circulatory death autotransplantation model; however, no benefit was demonstrated with prolonged storage, suggesting that any potential effects may be organ- and preservation solution-specific, as well as time-dependent. Larger studies are needed to draw more definitive conclusions.

## INTRODUCTION

Kidney transplantation remains the only curative treatment for patients with end-stage kidney failure. Static cold storage (SCS), largely unchanged >60 y, is a pillar of organ preservation owing to its simplicity. A flush of cold preservation solution followed by storage inside an ice-filled cooler has been the cornerstone of organ preservation. By lowering organ temperature, metabolism is slowed to limit energy and oxygen depletion, free-radical formation, and irreversible cell death. However, hypothermia does not preserve oxygen availability: residual metabolism rapidly depletes the limited oxygen reserves within the organ, driving a shift toward anaerobic pathways, progressive ATP depletion, and accumulating cellular injury that ultimately manifests as ischemia-reperfusion injury upon reperfusion.^1,2^ Furthermore, ice-based storage has been reported to lower temperatures into subzero ranges,^3-5^ raising the potential for freezing-related cellular and microvascular injury.^6,7^ In clinical practice, kidney SCS is usually limited to <24–36 h, given elevated rates of delayed graft function (DGF) and worse overall graft survival rates.^8-11^

Recently, pivotal preclinical studies have exposed the beneficial effects of shifting toward 10 °C storage of pig lungs when compared with classic SCS. Inspired by unexplored findings dating back to the late 1980s, 10 °C-stored lungs exhibited improved compliance, oxygenation, lower airway pressure, and better fluid management in animal and human trials.^12,13^ Additional groups have published comparable results, highlighting the reproducibility of 10 °C lung storage and its potential for semielective transplant while avoiding overnight shifts.^14,15^ While the mechanism remains to be fully elucidated, metabolomic studies reveal preservation of the tricarboxylic acid pathway and active β-oxidation, suggesting preserved mitochondrial function.^16^

As these data strengthen the protective role of 10 °C SCS in lung transplantation, interest across other organs has grown. Preclinical and clinical studies in cardiac transplantation align with the findings reported by lung transplant teams,^17,18^ and porcine livers stored at 10 °C have shown improved vascular resistance and a favorable bile quality profile.^19^ To our knowledge, no in vivo data report on outcomes of 10 °C SCS in kidney transplantation. Our goal was to determine whether 10 °C storage is beneficial for kidney preservation and transplantation compared with classical on-ice storage, using a porcine autotransplant model.

## MATERIALS AND METHODS

### Study Design and Protocol

The use of experimental animals for this study was approved by the institutional animal care and use committee of the University Healthcare Network under the Animal Use Protocol No. 3652. Intubation, central venous, and arterial line placement have been previously described^20^ and laparotomy, retrieval, and transplantation were performed as published by Kaths et al.^21^

**FIGURE 1.**
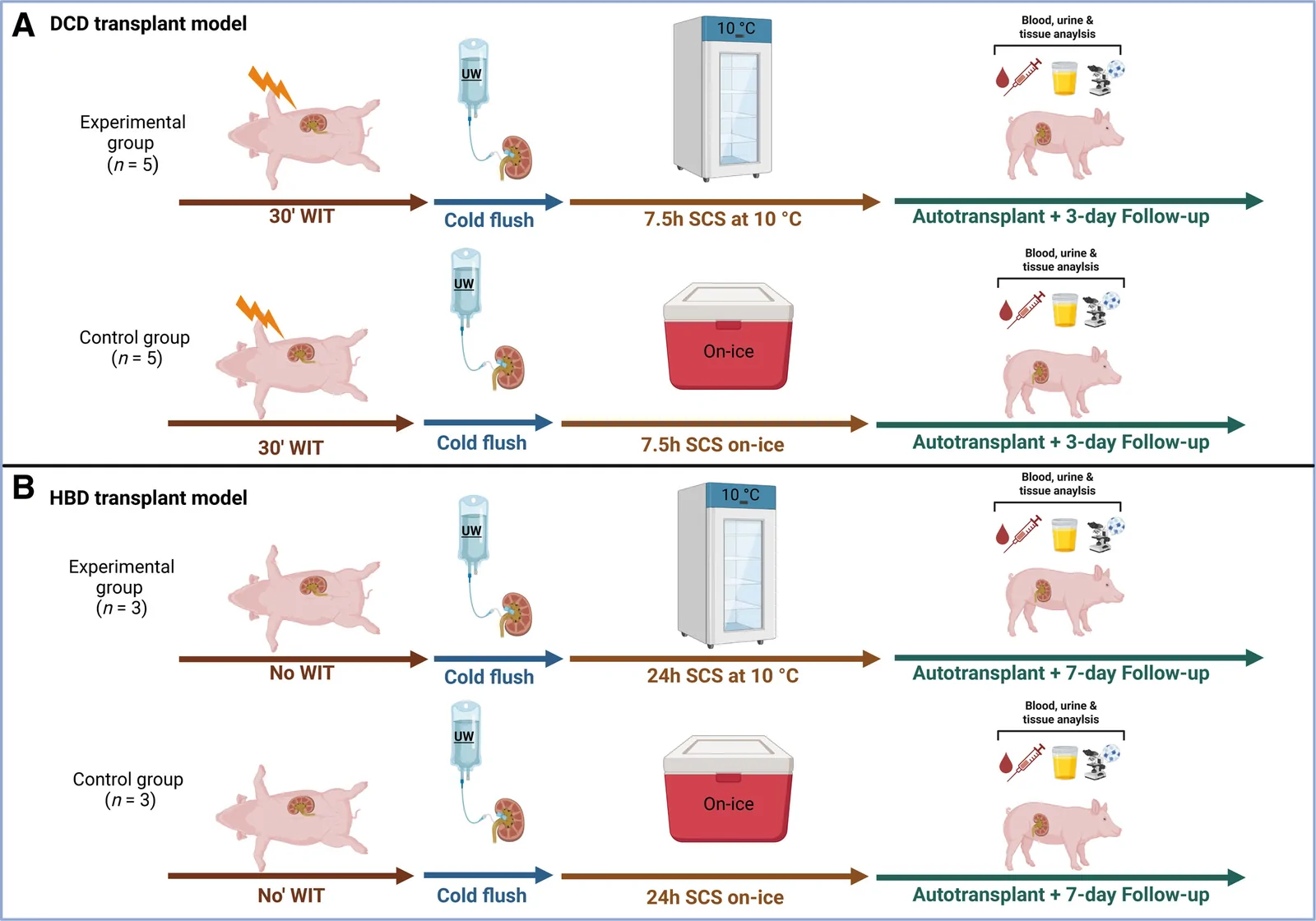
Study design. A, Outline for the DCD model. A total of 10 pigs were allocated to either 10 °C (n = 5) or on-ice SCS (n = 5). After contralateral nephrectomy and auto transplantation, subjects were followed for a 3-d period. B, Outline for the DBD autotransplant model. Three kidneys were retrieved with minimal warm ischemia, followed by cold flush and 24 h of storage at 10 °C. This was compared with a recent cohort of DBD kidneys stored conventionally on-ice for 24 h and followed up for 7 d after transplantation. HBD, heart beating donor; DCD, donation after circulatory death; SCS, static cold storage; UW, University of Wisconsin preservation solution; WIT, warm ischemia time.

In the donation after circulatory death (DCD) model, a unilateral hilum clamp was used to achieve 30 min of warm ischemia, followed by retrieval and cold flush with University of Wisconsin preservation solution (UW) storage solution (Bridge to Life, Northbrook, IL). Participants were randomized to graft storage at either 10 °C (n = 5) within a thermoelectric cooler (±0.5 °C accuracy) validated by other groups^13,22^ or inside an icebox with melting ice (n = 5), replicating conventional clinical practice in which grafts are stored without active temperature control, kept inside 2 sterile organ bags (CardioMed Supplies Inc., Lindsay, ON, Canada). After 7.5 h, grafts were transplanted and participants followed for 3 d. Animals received 1 g cefazolin and 500 mg/100 mL metronidazole twice daily throughout follow-up, with standardized parenteral hydration (Ringer’s lactate, 200 mL twice daily). The protocol is shown in Figure 1A.

To determine whether the effects of 10 °C storage vary by type of ischemic injury, a donation after brain death (DBD) model with minimal warm ischemia was developed. A 24-h preservation time was selected based on prior experience demonstrating moderate but consistent graft injury in this setting, providing sufficient range to detect a potential benefit,^23^ whereas a cold ischemia time (CIT) of 7.5–8 h would produce minimal postoperative injury, hindering the detection of any meaningful effect in a small cohort. In keeping with the 3R principles (Replacement, Reduction and Refinement),^24^ the 10 °C group (n = 3) was compared against a recently completed on-ice cohort (n = 3). Both DBD cohorts followed identical perioperative and intraoperative management protocols, anesthesia regimens, and surgical procedures, with the same postoperative care including twice daily antibiotic prophylaxis and hydration. Blood was sampled once daily in the morning, and participants were followed for 7 d after transplant.

### Blood and Urine Assessment

Blood gas assessment was performed with a GEM premier 5000 (Werfen, Bedford, MA). Samples were centrifuged at 10 000 revolutions per minute for 10 min at 4 °C, snap-frozen in liquid nitrogen, and stored at –80 °C. Biochemical analysis used Piccolo Xpress (Abaxis, Union City, CA). Neutrophil gelatin-associated lipocalin (NGAL) was measured using a porcine lipocalin-2/NGAL ELISA Kit (Novus Biologicals, Toronto, ON, Canada), and thiobarbituric acid reactive substances (TBARS), a product of lipid peroxidation, were assessed (R&D Systems, Minneapolis, MN), both per manufacturer’s instructions.

Urine was obtained by bladder puncture before nephrectomy and at sacrifice for spot analysis. Pigs were placed in a metabolic cage the day before procedures and on postoperative day (POD) 3 for 24-h collection. Urine samples were analyzed by the Toronto General Hospital core laboratory. Creatinine clearance ([creatinine_urine_] volume_urine_/[creatinine_plasma_]) and fractional excretion of sodium (FeNa; 100 × [creatinine_plasma_ × sodium_urine_]/[sodium_plasma_ × creatinine_urine_]) were calculated. Since dialysis and the diagnosis of DGF are not feasible in porcine models, we used severe renal dysfunction (SRD) as a surrogate, as defined and validated by Urbanellis et al,^25,26^ who addressed the challenges of mimicking DGF in these models and the limitations of available surrogates. SRD was defined as serum potassium of 6.0 mmol/L or greater with a 24-h urine output of 500 mL or less. While prior studies assessed this on the morning of POD 4, our latest measurement was obtained at 72 h postreperfusion (evening POD 3); sufficiently close to warrant the same definition and serving as an adequate surrogate for clinically significant graft dysfunction in this model.

### Histology

Tissue samples were obtained under general anesthesia at sacrifice. Three wedge biopsies were taken with a sharp N15 blade from the upper pole, lower pole, and midsection to account for heterogeneity in warm ischemic damage. These were fixed in buffered formalin for 24 h and transferred to 70% alcohol, then processed at Toronto General Hospital. Paraffin-embedded 3 µm sections were stained with Periodic Acid-Schiff and scanned at ×20 using an Aperio CS2 (Leica Biosystems, Nussloch, Germany). Tubular injury (vacuolization, brush-border loss, sloughing, and luminal debris) and interstitial inflammation were graded by a blinded pathologist (R.J.) on a 0–3 scale with 0.25 intervals, adapted from Goujon et al.^27^ Additional samples were obtained at baseline, after storage (before transplantation), and 30 min after reperfusion with an 18 gauge × 16 cm biopsy gun (BD, Mississauga, ON, Canada) and processed as described.

### Statistical Analysis

Continuous results were expressed as mean and SD or median and interquartile range (IQR) when appropriate. Unpaired *t* tests compared continuous variables. The area under the curve (AUC) was calculated individually for repeated measures and was compared between groups by unpaired *t* test. Urine output, creatinine clearance, and FeNa were analyzed by mixed-model analysis with post hoc Holm-Šídák correction for multiple comparisons. Statistical significance was defined at *P* < 0.05. GraphPad Prism v10.4.27 was used for calculations and graphs.

## RESULTS

### DCD Model

#### Baseline Characteristics and Outcomes

No significant differences were identified between groups in pig and graft weights, warm ischemia times (WITs), CIT, or anastomotic times (Table 1). All participants in the 10 °C group reached the prespecified endpoint, while 1 on-ice participants was sacrificed earlier (64 h after transplant) because of persistently severe hyperkalemia. Necropsy showed patent vascular structures with an adequately perfused kidney and an unobstructed ureteral anastomosis with an indwelling stent. This participant met SRD criteria, with a potassium of 7.1 mmol/L at sacrifice and no urine output during follow-up. In total, 2 (40%) of the on-ice cohort met both SRD criteria, while no participant in the 10 °C group did.

**TABLE 1.** Baseline and perioperative variables for the 30-min warm ischemia and the 24-h static cold storage models

Variable	30 min WIT + 7.5 h SCS			24 h SCS		
	10 °C (n = 5)	On-ice (n = 5)	*P*	10 °C (n = 3)	On-ice (n = 3)	*P*
Pig weight, kg, mean ± SD	29.3 ± 1.5	31.4 ± 2.7	0.15	29.4 ± 1.8	28.9 ± 1.6	0.74
Kidney weight, grams, mean ± SD	93.4 ± 9.0	92.8 ± 5.3	0.90	99.7 ± 20.1	90.0 ± 7.0	0.48
Weight change after storage, %, mean ± SD	–9.4% ± 5.8%	–3.0% ± 5.6%	0.11	–6.7% ± 5.9%	+1.8% ± 6.9%	0.23
Warm ischemia time, min, mean ± SD	31.8 ± 1.8	32.4 ± 2.3	0.66	N/A	N/A	N/A
Anastomosis time, min, mean ± SD	23.2 ± 1.5	24.6 ± 2.4	0.30	23.7 ± 1.2	25.0 ± 2.0	0.37
Preservation time, mean ± SD	468 ± 8.0 min	461.6 ± 6.9 min	0.21	24.5 ± 0.04 h	24.6 ± 0.7 h	0.53

Unpaired *t* tests were performed to compare groups.

N/A, not available; SCS, static cold storage; WIT, warm ischemia time.

#### Posttransplant Graft Function and Injury Markers

**FIGURE 2.**
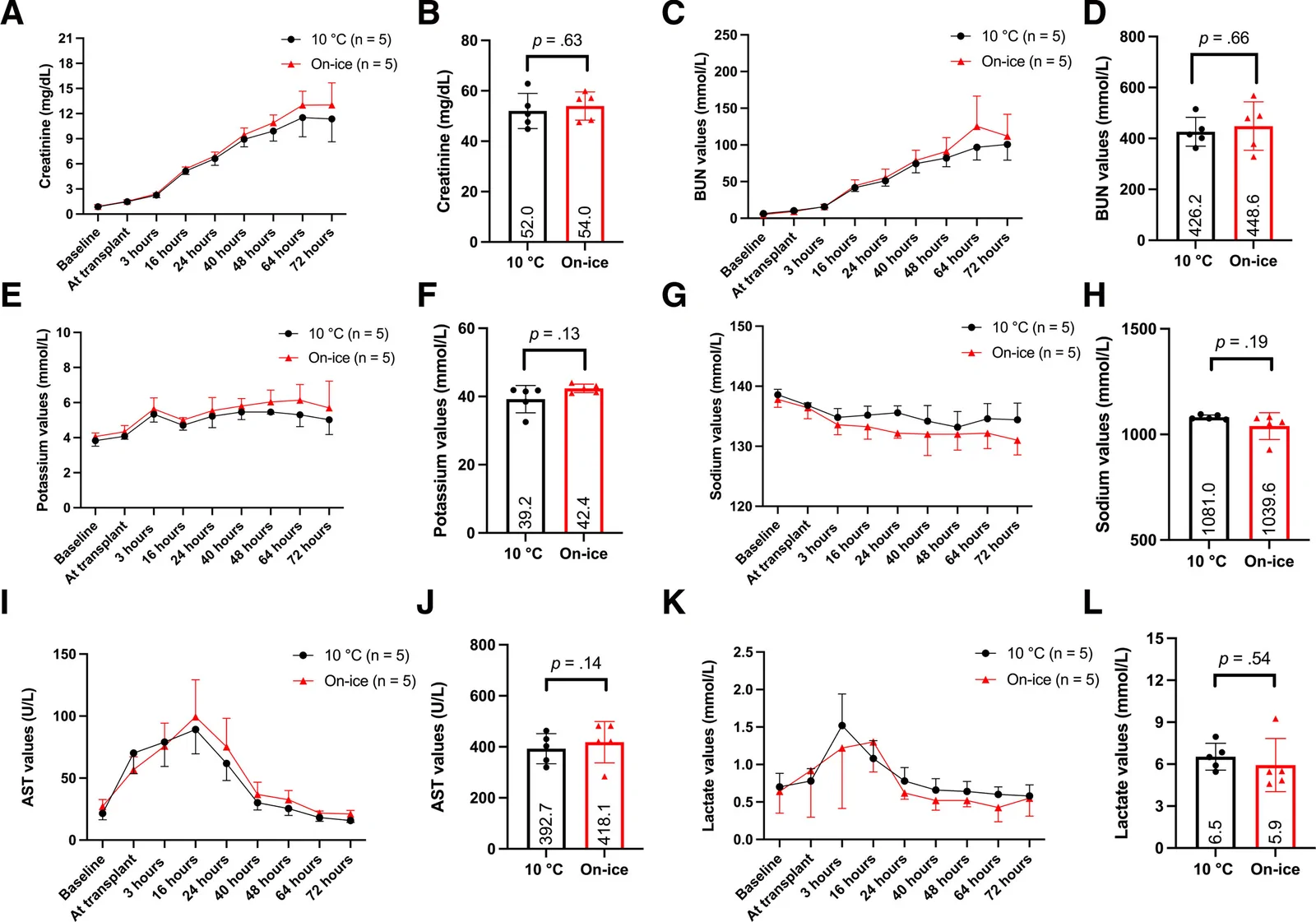
Serum marker levels of 10 °C (n = 5) vs on-ice (n = 5) 30 min warm ischemia time (WIT) and 7.5 h static cold storage (SCS) experiments: Creatinine levels (A), creatinine AUC (B), BUN levels (C), BUN AUC (D), potassium levels (E), potassium AUC (F), sodium levels (G), sodium AUC (H), AST levels (I), AST AUC (J), lactate levels (K), and lactate AUC (L) before, during, and 3 d after transplantation. Error bars represent SD. AST, aspartate aminotransferase; AUC, area under the curve; BUN, blood urea nitrogen.

Creatinine peaked at 64 h postreperfusion in the 10 °C group (11.5 ± 2.3 mg/dL) and at 72 h in the on-ice group (13.0 ± 2.6 mg/dL), without significant differences (*P* = 0.63; Figure 2A and B). Blood urea nitrogen (BUN) values followed a similar trend, peaking at POD 3 (64–72 h) in both groups with no significant differences (*P* = 0.66; Figure 2C and D). Potassium remained elevated in both groups, with the 10 °C group trending lower (*P* = 0.13; Figure 2E and F), and 10 °C storage trended toward higher serum sodium (*P* = 0.19; Figure 2G and H). Aspartate aminotransferase (Figure 2I and J) peaked at 16 h posttransplant (89.2 ± 19.6 U/L for the 10 °C group versus 99.4 ± 29.9 U/L on-ice), returning to baseline by POD 3 in both groups (*P* = 0.14). Lactate was consistent between groups (*P* = 0.96; Figure 2K and L). **Table S1** (**SDC**, https://links.lww.com/TXD/A890) provides the reference value for all serum parameters based on baseline samples from 70 Yorkshire pigs in the last 2 y.

#### Urine Analysis

**FIGURE 3.**
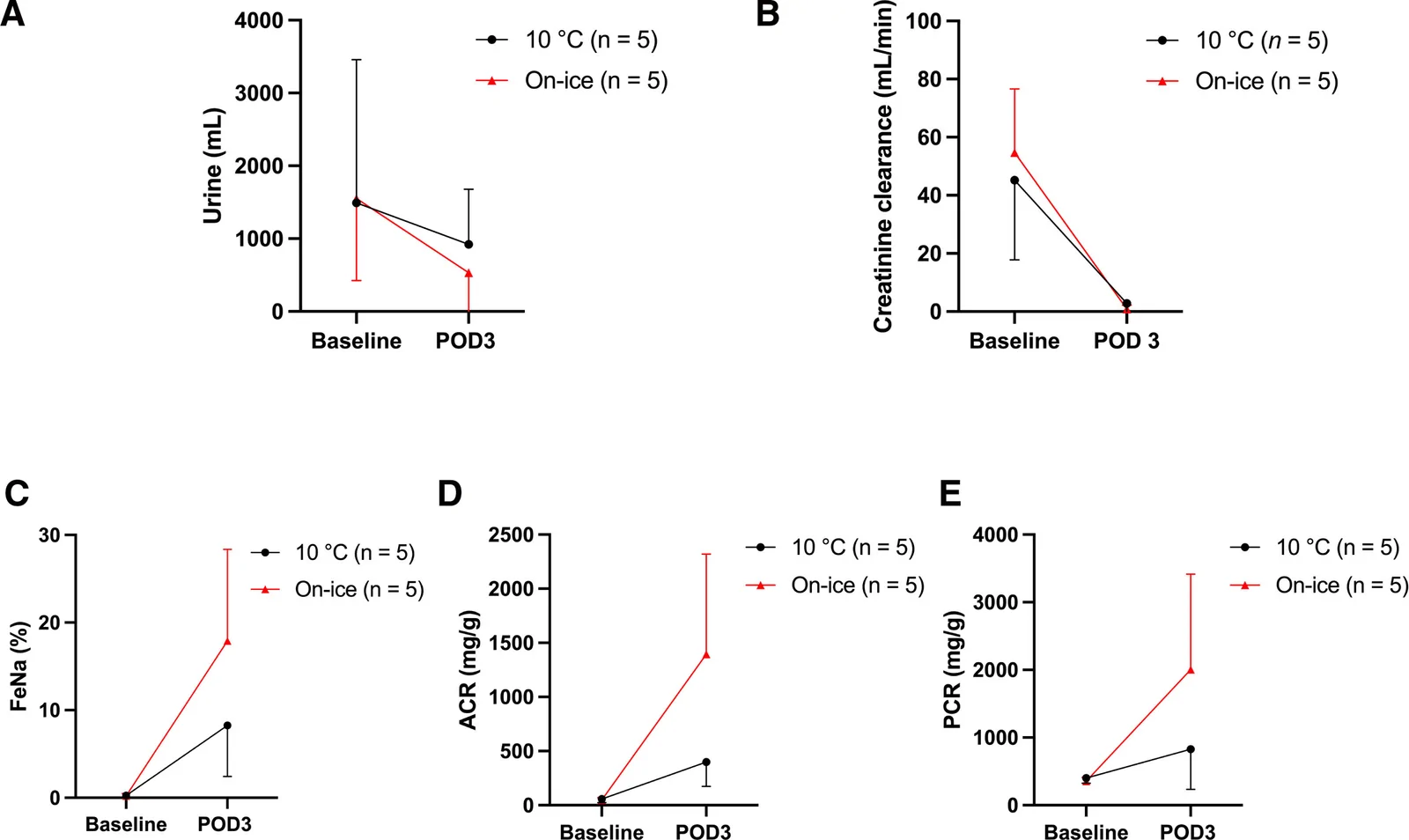
Urine analysis for the donation after circulatory death (DCD) model. Kidneys were subjected to 30 min of warm ischemia and allocated to 7.5 h of static cold storage at either 10 °C (n = 5) or on-ice (n = 5). A, 24-h urinary output in mL. B, Creatinine clearance in mL/min. C, FeNa as a percentage. D and E, ACR and PCR. Error bars show the SD. Multiple unpaired *t* tests were used to compare groups within each timepoint, corrected by the Holm-Šídák method. No significant differences were observed after the correction. ACR, albumin-to-creatinine ratio; FeNa, fractional excretion of sodium; PCR, protein-to-creatinine ratio; POD, postoperative day.

Baseline 24-h urine output was similar between groups, with medians of 700 mL (IQR, 450–2925 mL) for 10 °C and 1800 mL (IQR, 375–2600 mL) for on-ice. Postoperatively, median output was higher in the 10 °C group (850 mL [IQR, 275–1600 mL] versus 100 mL [IQR, 0–1275 mL]) without significant differences (*P* = 0.77; Figure 3A). Creatinine clearance fell from 35 mL/min (IQR, 21.2–74.4 mL/min) at baseline to 0.8 mL/min (0.4–6.1 mL/min) at POD 3 in the 10 °C group, with a similar decrease on-ice (35 mL/min; 21.2–74.4 to 0.01 mL/min; 0.001–2.26; Figure 3B). These changes were significant over time (*P* < 0.005), but not between storage methods (*P* = 0.64).

Baseline FeNa was low in both groups (0.2% [IQR, 0.10%–0.45% for 10 °C] versus 0.1% [IQR, 0.1%–0.4% for on-ice]). Posttransplant FeNa was higher on-ice (19.9% [IQR, 8.2%–26.7%] than 10 °C [7%; IQR, 2.95%–14.20%]); overall group differences were not significant (*P* = 0.09), but post hoc testing showed a significant difference at POD 3 (*P* = 0.04; Figure 3C). The albumin-to-creatinine ratio changed significantly over time, with on-ice kidneys markedly higher than 10 °C at POD 3 (1394.8 ± 925.4 mg/g versus 398.7 ± 224.5 mg/g; time × group interaction: *F*(1, 16) = 5.544; *P* = 0.03). Post hoc Šídák-corrected comparisons showed no difference at baseline (*P* = 0.99) but a significant difference at POD3 (mean difference, –996.2; 95% confidence interval, –1740 to –252.8; *P* = 0.0089). Protein-to-creatinine ratio followed the same trend without reaching significance (*F*(1, 16) = 2.716; *P* = 0.09), with borderline significance at POD 3 (mean difference, –1175; 95% confidence interval, –2372 to 20.79; *P* = 0.05; Figure 3E).

#### Tissue Injury and Inflammation

**FIGURE 4.**
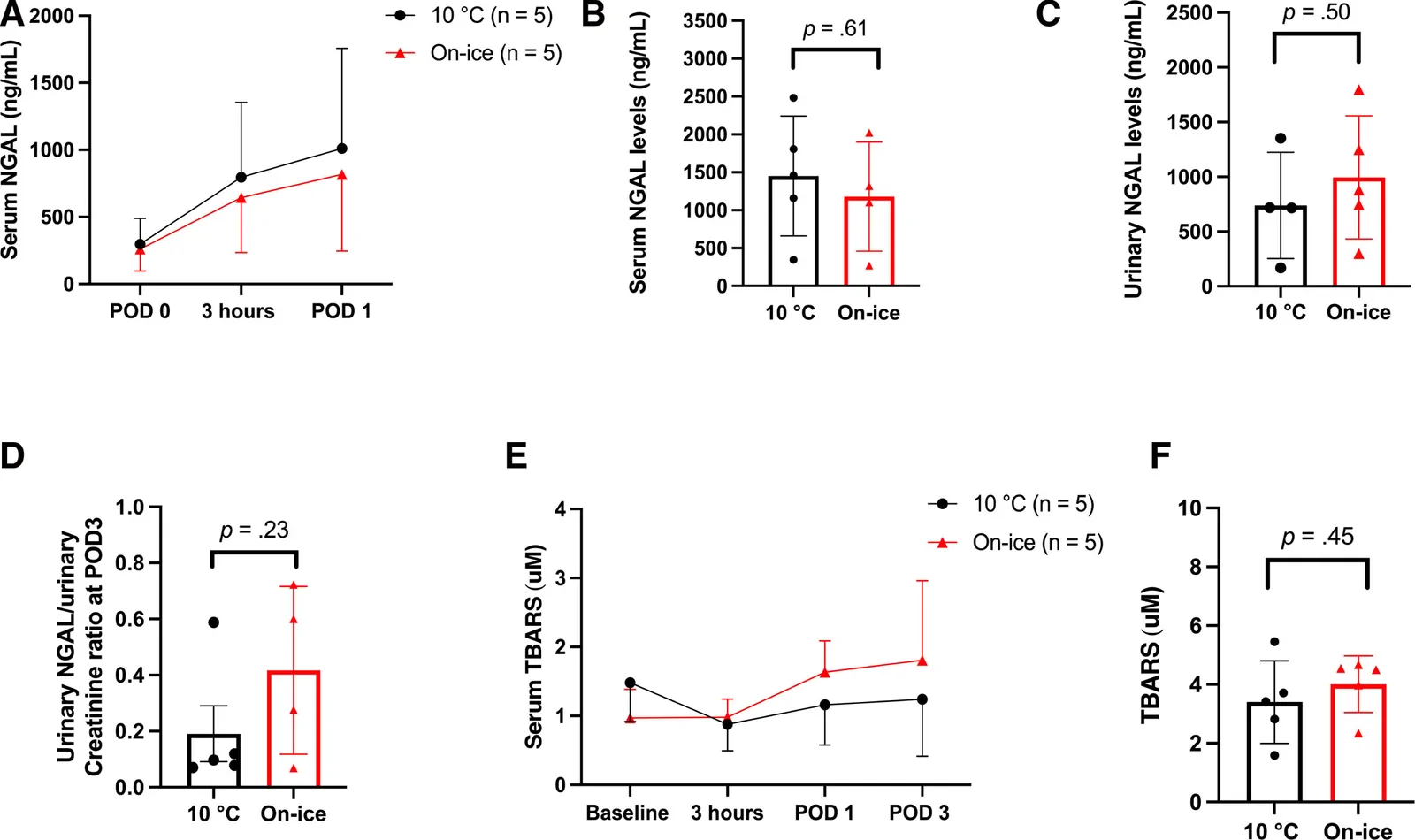
NGAL and TBARS were assessed. For NGAL, 3 serum samples (baseline, 3 h postreperfusion, and POD 3) were analyzed (A), and the AUC was compared in (B). A urine sample at POD 3 was obtained for NGAL assessment (C), and the urinary NGAL/urinary creatinine ratio was calculated and compared between groups via an unpaired *t* test (D). Four serum samples (baseline, 3 h postreperfusion, POD 1, and POD 3) were processed to assess TBARS levels, as seen in (E) and (F), corresponding to the levels throughout the different time points, and the AUC, respectively. No significant differences were identified. AUC, area under the curve; NGAL, neutrophil gelatinase-associated lipocalin; POD, postoperative day; TBARS, thiobarbituric acid reactive substances.

NGAL was analyzed in serum at baseline, 3 h postreperfusion, and POD 1, peaking at POD 1 in both groups (1011.4 ± 746.5 ng/mL for 10 °C versus 816.9 ± 570.4 ng/mL on-ice; Figure 4A), with no significant differences (Figure 4B). Urinary NGAL at POD3 trended lower in the 10 °C group (738.3 ± 485.2 ng/mL versus 994.7 ± 563.1 ng/mL; *P* = 0.50; Figure 4C), a difference highlighted by the NGAL/urinary creatinine ratio (0.19 ± 0.22 versus 0.42 ± 0.30; *P* = 0.23; Figure 4D). The TBARS assay, measuring serum malondialdehyde as an index of lipid peroxidation, was performed at baseline, 3 h postreperfusion, POD 1, and POD 3, peaking at POD 3 in both groups (1.2 ± 0.8 versus 1.8 ± 1.2 for 10 °C versus on-ice; Figure 4E). Values were consistently lower at 10 °C but did not reach statistical significance by AUC comparison (*P* = 0.24; Figure 4F).

**FIGURE 5.**
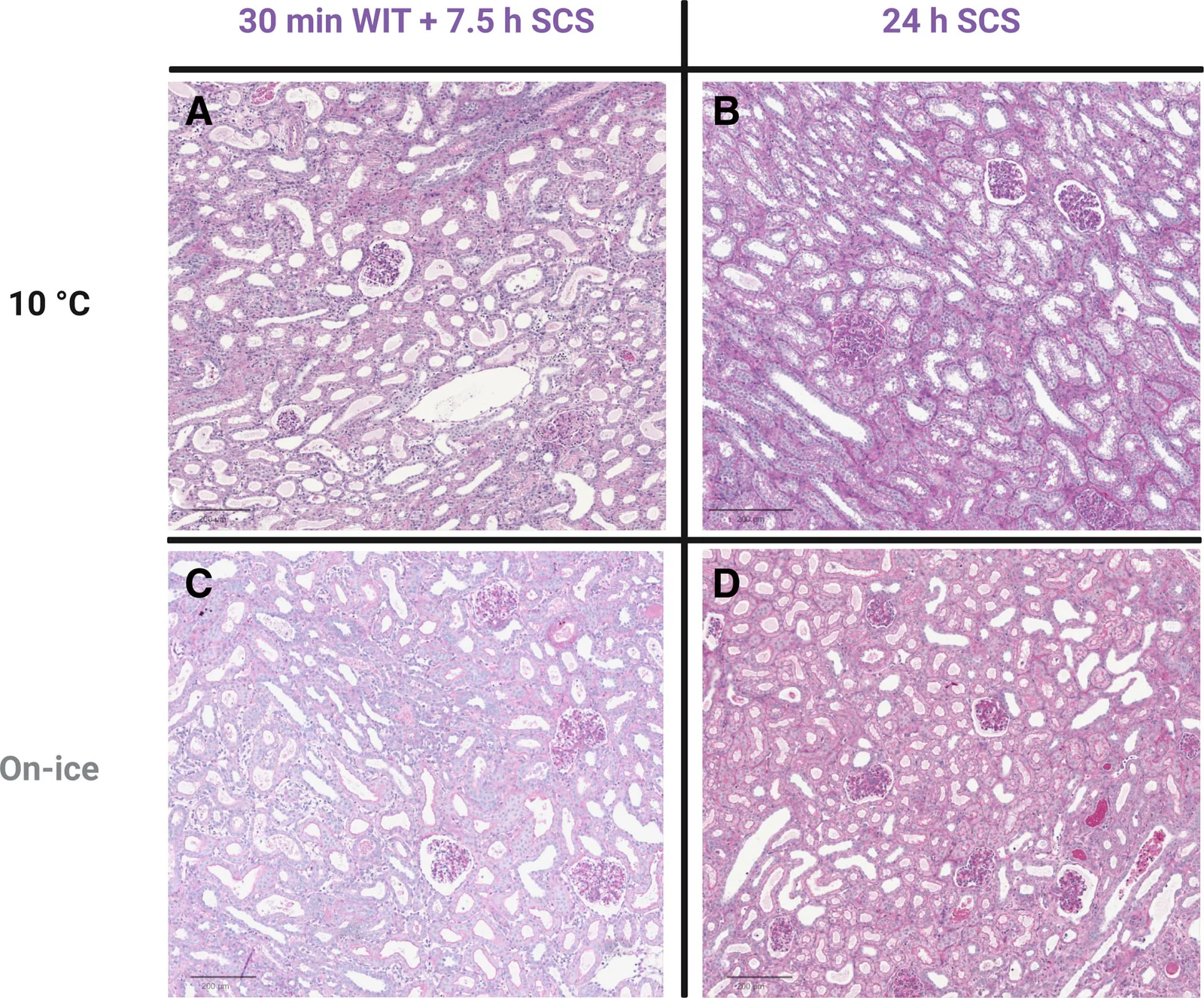
Representative histological images for both sets of experiments. 10 °C (upper row) and on-ice (lower row) have been grouped by protocol: 30-min warm ischemia + 7.5 h SCS in (A) and (C), and the minimal warm ischemia with 24 h SCS model in (B) and (D). SCS, static cold storage; WIT, warm ischemia time.

Tissue samples obtained at termination were assessed for tubular injury and interstitial inflammation by a pathologist masked to group and clinical outcomes, with per-experiment averages compared between groups. Mean tubular injury was significantly lower in the 10 °C group (1.69 ± 0.37 versus 1.97 ± 0.29; *P* = 0.03), as was interstitial inflammation (1.19 ± 0.46 versus 1.63 ± 0.29; *P* = 0.005). Results are shown in Table 2, with representative macroscopic and microscopic images in Figure 5.

**TABLE 2.** Results for histological scoring at 10 °C and on-ice storage upon termination at postoperative day 3 for the DCD model and postoperative day 7 for the DBD model

Outcome	30 min WIT + 7.5 h SCS			24 h SCS		
	10 °C (n = 5)	On-ice (n = 5)	*P*	10 °C (n = 3)	On-ice (n = 3)	*P*
Tubular injury	1.69 ± 0.37	1.97 ± 0.29	0.03	2 (1–2.5)	2 (0.5–2)	0.77
Interstitial inflammation	1.19 ± 0.46	1.63 ± 0.29	0.005	2 (0.5–2.5)	1.5 (1.5–3)	0.69

*P* values correspond to unpaired *t* tests.

DBD, donation after brain death; DCD, donation after circulatory death; SCS, static cold storage; WIT, warm ischemia time.

### Twenty-four-hour SCS Experiments

**FIGURE 6.**
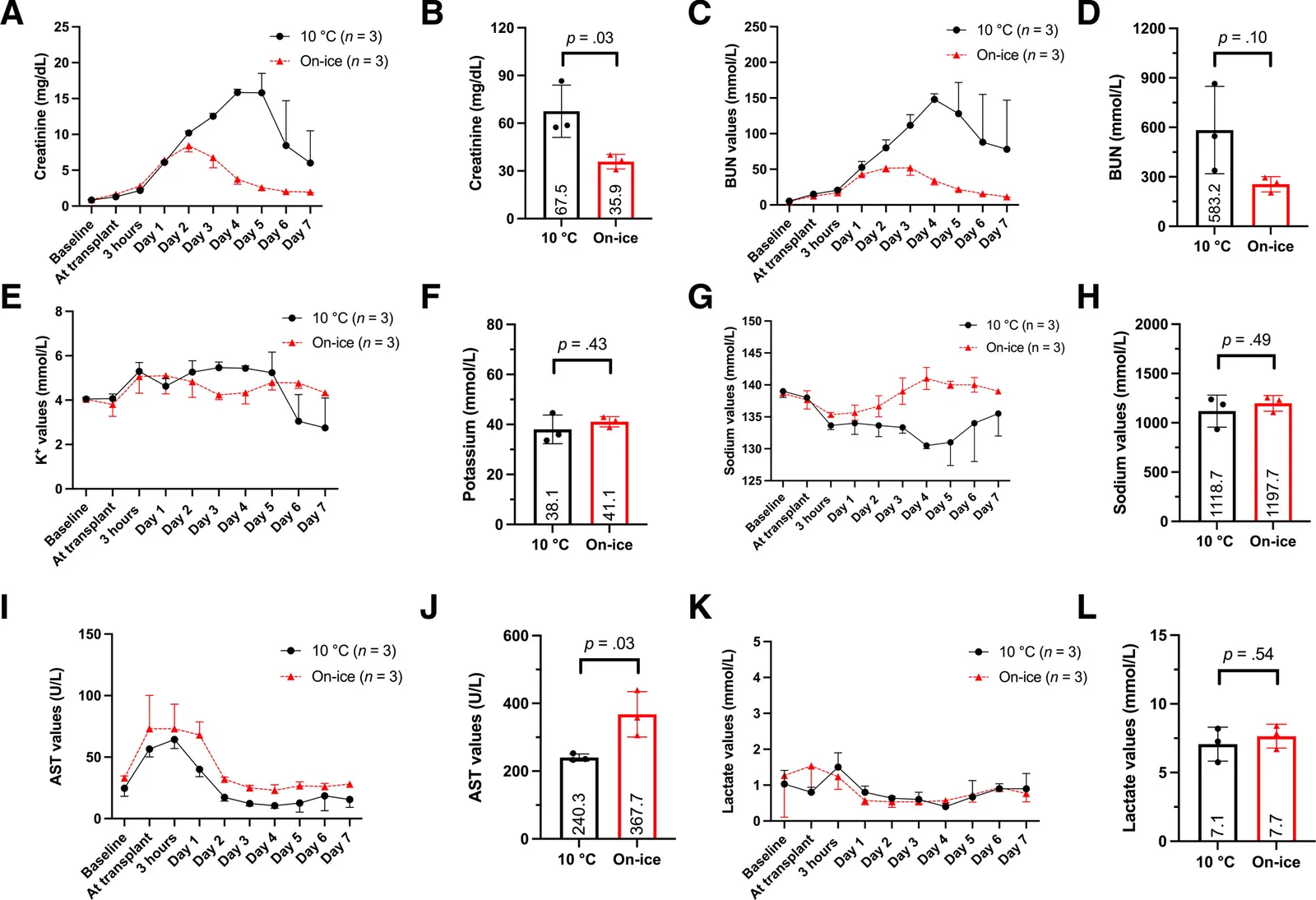
Serum marker levels of 10 °C (n = 3) vs on-ice (n = 3) for the 24 h static cold storage (SCS) experiments: Creatinine levels (A), creatinine AUC (B), BUN levels (C), BUN AUC (D), potassium levels (E), potassium AUC (F), sodium levels (G), sodium AUC (H), AST levels (I), AST AUC (J), lactate levels (K) and lactate AUC (L) before, during, and 7 d after transplantation. Error bars represent SD. AST, aspartate aminotransferase; AUC, area under the curve; BUN, blood urea nitrogen.

To determine whether 10 °C storage has a different impact depending on ischemia type, we investigated a DBD model of minimal warm ischemia, prolonged SCS (24 h), and autotransplantation with contralateral nephrectomy. Storage at 10 °C for 24 h was compared with conventional icebox SCS (n = 3 per group), replenishing ice levels every 12 h. Participants were followed for 7 d after transplantation (Figure 1B), with comparable baseline values between groups (Table 1). One early termination occurred at POD 5 in the 10 °C group because of persistently elevated creatinine and BUN; necropsy was unremarkable, with a well-perfused kidney, unobstructed ureteral anastomosis, and patent vasculature. Creatinine values were comparable between groups until POD 3, peaking at POD 5 in the 10 °C group (15.8 ± 8.8 mg/dL), versus POD 2 on-ice (8.4 ± 1.5 mg/dL; Figure 6A), with the AUC significantly favoring on-ice (*P* = 0.03; Figure 6B). BUN was congruent, peaking at POD 4 in the 10 °C cohort (148.0 ± 11.3 mg/dL), versus POD 2 on-ice (51.7 ± 18.0 mg/dL; *P* = 0.10; Figure 6C and D). Potassium fluctuated in both groups without reaching significance (*P* = 0.69; Figure 6E and F), and sodium also favored on-ice storage (*P* = 0.49; Figure 6G and H). Aspartate aminotransferase peak and AUC were significantly lower at 10 °C (64.3 ± 7.2 versus 73.0 ± 20.1; *P* = 0.03; Figure 6I and J). Lactate was consistent throughout groups and time points (*P* = 0.54; Figure 6K and L).

**FIGURE 7.**
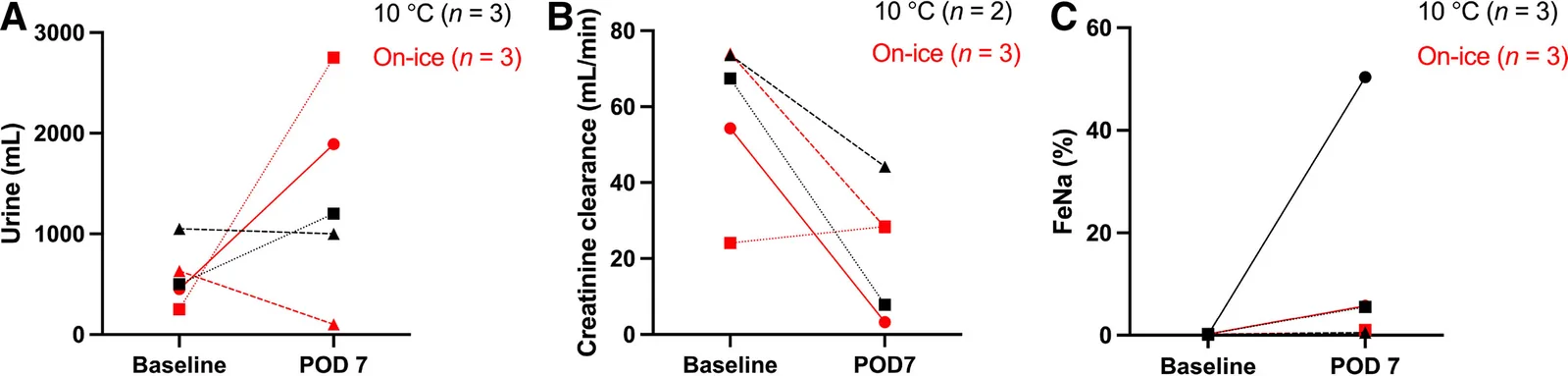
Urine analysis of the 24 h static cold storage (SCS) autotransplant model. Kidneys were stored at either 10 °C (n = 3) or on-ice (n = 3) after a minimal warm ischemia retrieval for a period of 24 h. Urine samples were taken at baseline and at POD 7. A, Urinary output. B, Creatinine clearance. C, FeNa. Error bars correspond to SD. No significant differences were identified between the groups. FeNa, fractional excretion of sodium; POD, postoperative day.

Twenty-four-hour urine output was similar between groups at baseline (775 mL [IQR, 500–1050 mL] versus 450 mL [IQR, 250–630 mL]) and POD 7 (1100 mL [IQR, 1000–1200 mL] versus 1890 mL [100–2750 mL]; *P* = 0.89; Figure 7A). Median creatinine clearance showed a significant worsening from baseline to POD 7 without between-group differences (baseline, 70.5 mL/min [IQR, 67.4–73.6 mL/min] versus 54.3 mL/min [IQR, 24.1–74.1 mL/min]; *P* = 0.31 and POD 7, 26.0 mL/min [IQR, 7.8–44.2 mL/min] versus 28.2 mL/min [IQR, 3.2–28.4 mL/min]; Figure 7B), in both initial and post hoc analyses. Median FeNa worsened at sacrifice in both groups, trending worse at 10 °C (5.6% [IQR, 0.5%–50.4%] versus 1% [IQR, 0.5%–5.8%]; *P* = 0.39; Figure 7C). Tissue analysis showed comparable tubular injury (2.0 [IQR, 1–2.5] versus 2.0 [IQR, 0.5–2]) and interstitial inflammation (2.0 [IQR, 0.5–2.5] versus 1.5 [IQR, 1.5–3.0]), without statistical significance (Table 2).

## DISCUSSION

This is the first report comparing on-ice to 10 °C storage in a porcine kidney autotransplant model. A 2-modeled approach was used to simulate real-world deceased donation scenarios, given the lack of evidence on 10 °C in kidney transplantation.

The DCD model was designed to simulate the fast-growing practice of DCD donation, which accounted for >40% of all deceased donors in the United States in 2024.^28^ An 8-h storage time aligns with common practice in many regions, and prior studies from our group have shown that 30 min of warm ischemia followed by 8 h of cold ischemia generates clinically significant graft damage^29,30^ that could potentially be mitigated by 10 °C storage. While creatinine, BUN, potassium, and sodium values, 24-h urinary output, and FeNa showed trends favoring 10 °C storage, numerical differences between groups were modest. Notably, no participants in the 10 °C group met SRD criteria, while 2 in the on-ice group did, suggesting the model retains sufficient sensitivity to detect clinically meaningful differences. These findings are strengthened by low intergroup variability, and peak creatinine observed in our SCS control group (11.5 ± 2.3 mg/dL at 64 h) closely mirrors the 11.3 ± 2.0 mg/dL reported by Kaths et al^29^ at POD 3 in a comparable DCD/SCS cohort, supporting the reproducibility of our model across a nearly decade-long interval. The 3-d follow-up, intended to capture histological differences as per previous reports,^29,30^ significantly favored 10 °C, although unaccompanied by strong clinical benefit and should be interpreted cautiously. Ancillary NGAL measurements in urine and serum and TBARS assays to detect lipid peroxidation, a surrogate of mitochondrial health, showed no benefit from 10 °C storage. Taken together, these results do not allow firm conclusions regarding the benefit of 10 °C storage over on-ice storage in our porcine model, given the small sample size and marginal differences in this initial study.

Building on studies demonstrating that 10 °C storage protected organ health following minimal warm ischemia and prolonged CIT,^12^ we hypothesized that its effects may differ by type of injury. To evaluate this, we developed a second model using minimal WIT with prolonged on-ice SCS (24 h). In keeping with the reduction principle,^24^ the experimental group was compared against a cohort recently completed by the same team within the preceding 3 mo.^23^ This was further justified by our experience demonstrating that shorter CIT in our DBD porcine model produced minimal injury, making the existing 24-h cohort the most appropriate comparator to detect a benefit of 10 °C storage. While a small cohort (n = 3), experiments were conducted in a strict manner with comparable preoperative and intraoperative variables.

No benefit was observed from increasing the storage temperature to 10 °C, and worse creatinine values were seen following transplantation. While the design was driven by pragmatic and ethical considerations, we acknowledge that comparing 2 models with different donor types, preservation durations, and follow-up limits complicates cross-cohort interpretation. Reasons for the lack of benefit with prolonged storage remain uncertain, with a possibility that 10 °C storage may hasten cellular energy depletion over extended ischemic times, leading to greater cellular damage upon reperfusion.

Organ-specific factors may account for the discrepancy between other reports and our results. Pig lungs stored at 10 °C expressed mitochondrial health-related metabolites such as itaconate and glutamate, alongside reduced circulating free mitochondrial DNA in perfusate, suggesting preserved mitochondrial function.^12^ Mitochondrial respiration was increased, pointing to higher oxygen reserves to sustain metabolic activity. Unlike other organs, lungs are insufflated and stored with oxygen, permitting a degree of aerobic metabolism.^31^ Furthermore, kidneys account for approximately 6% of total body oxygen consumption while representing only 0.5% of body mass, compared with 4% and 0.9%, respectively, for lungs,^32^ reflecting a substantially higher mass-corrected metabolic demand. The greater metabolic activity and absence of supplemental oxygen may put kidneys at higher risk for energy depletion at 10 °C and contribute to the differences observed relative to lung preservation.^33^ However, organ-specific metabolic differences alone may not fully explain the discrepancy.

Tracy et al^19^ assessed porcine livers subject to 10 min of WIT followed by 10 h of SCS at 10 °C or on-ice (n = 5 per group) with subsequent 4-h normothermic machine perfusion assessment. While traditional markers, including lactate clearance and transaminases, did not differ, 10 °C storage enhanced hepatic artery vasoresponsiveness, portal venous resistance, bile production, pH, and bicarbonate. Untargeted metabolomics revealed preserved mitochondrial function in 10 °C livers, including reduced anaerobic metabolism markers, maintenance of electron transport chain function, and less DNA damage on Terminal deoxynucleotidyl transferase dUTP nick end labeling staining, suggesting hepatocellular benefit beyond conventional serum markers. Mechanistically, the authors propose that methylglutaric acid during ice storage drives Na/K-ATPase inhibition upon reperfusion, corroborating the pivotal role of ATPase preservation described by Ali et al.^12^ While encouraging, these findings were obtained ex vivo without transplantation, limiting translational applicability. Nonetheless, the convergence of vascular, biliary, mitochondrial, and ATPase-related findings across lung and liver preservation contrasts with our kidney results, suggesting that factors beyond storage temperature may govern the magnitude of benefit observed across different organs.

Literature on 10 °C heart preservation, however, challenges a purely organ-specific explanation. Accounting for roughly 0.4% of total body mass while consuming 11% of total body oxygen,^32^ the heart exceeds the kidney’s metabolic demands. Despite this, an analysis of 52 hearts stored at 10 °C against propensity-matched controls found reduced intraoperative lactate levels and improved cardiac index at 72 h, with comparable rates of severe primary graft dysfunction.^34^ A subsequent study of DCD hearts following normothermic thoracoabdominal regional perfusion demonstrated lower primary graft dysfunction rates and vasoactive-inotrope scores, shorter ICU stay, and improved 6-mo survival with 10 °C.^35^ Although observational and nonrandomized, these benefits point toward a factor beyond intrinsic metabolic rate.

Preservation solution composition may account for this discrepancy. By flushing 10 °C-stored lungs with the ATPase inhibitor ouabain before 36-h storage, Ali et al showed significantly worse airway pressures, compliance, and oxygenation, alongside greater weight gain, metabolic derangement, and mitochondrial DNA release during ex vivo perfusion. These findings underscore that the benefits of 10 °C preservation are not temperature-dependent alone, but contingent on preserved ATPase function. In this context, UW solution, with a potassium concentration of approximately 120 mEq/L, virtually abolishes the electrochemical gradient required for ATPase activity, rendering the pump inactive despite a favorable storage temperature. Extracellular-type solutions such as Perfadex, Celsior, or Del Nido cardioplegia, which maintain a physiological ionic gradient, may provide a more permissive environment for residual ATPase activity at 10 °C, a condition that lungs and hearts benefit from given their preservation solutions. Whether substituting UW for an extracellular type solution could unlock the benefit of 10 °C storage in kidney transplantation remains a compelling question.

Our DCD model showed a general trend of protection with 10 °C storage across main outcomes, although these improvements were not statistically significant in most cases. These results must be interpreted with caution: while minimal clinical improvement is in line with published data on other organs, it is insufficient to categorically demonstrate the superiority of 10 °C storage. Discrepancies may reflect organ-specific, solution-specific, and model-specific factors. Lung and liver preclinical data derive from ex vivo settings only, and while a clinical study showed safety in the prolongation of times in lung and heart transplantation using 10 °C storage, these studies were not designed to show superiority.

Our study has several limitations. The low number of participants in each group, inherent to large-animal models, is the first limitation; nonetheless, the consistency of intergroup preoperative and intraoperative variables strengthens the findings. Our relatively short follow-up time was designed to capture significant histological changes, which was achieved, and prior experience from our group indicates that POD 3 is the point at which interventions start to show their effect through creatinine reduction. In our cohort, the 3-d endpoint preceded the full recovery in most animals, particularly in the 10 °C group, where creatinine trajectories at sacrifice suggested ongoing improvement.

A further limitation concerns the interpretation of urinary parameters. Although widely used, metabolic cages are well-documented sources of error, including cross-contamination with excreta and food and inadvertent sample loss.^36^ In our model, pigs have unrestricted access to water per institutional ethics protocols mandating adequate hydration and comfort postoperatively; while essential for animal well-being, this frequently causes spillage from the water tray into the urine tray, diluting samples. Other sources of error include the absorption of urine by food pellets dropped in the collection tray, inadvertent discarding of samples because of staff communication gaps, and tray overflow in cases of extreme diuresis. Despite rigorous measures to minimize these confounders, complete elimination is challenging, and these factors may contribute to overestimation or underestimation of creatinine clearance and related parameters, which should be interpreted with caution.

While mechanistic theories draw on mitochondrial health and Na/K-ATPase activity to explain the benefits of 10 °C, the present study did not assess mitochondrial metabolites or ATPase activity, as it was designed as an in vivo functional and histological assessment, and such analyses were beyond its scope. A dedicated mechanistic investigation incorporating mitochondrial metabolomics and ATPase activity profiling would constitute a meaningful future contribution. Finally, the 2-model design does not allow for the determination of the maximum safe CIT for DCD kidneys at 10 °C, nor a direct comparison between DCD and DBD kidneys under equivalent conditions, both important directions for future study.

In conclusion, this study is the first comparing on-ice and 10 °C storage of kidney grafts in an autotransplant model, showing modest functional improvement with short DCD storage and no demonstrable benefit with prolonged DBD storage. Whether benefits extend to prolonged storage remains unclear. Given the growing enthusiasm for 10 °C storage, further preclinical studies are needed to establish its effects on graft function and the factors modulating them.

## Supplementary Material


