# Supplementary material for: 10 °C Storage for the Preservation of Pig Kidney Grafts: A Porcine Autotransplant Model
Source: Transplant Direct. 2026 Aug 21;12(9):e1993. doi: 10.1097/TXD.0000000000001993 (PMC13502862; doi:10.1097/TXD.0000000000001993)
Supplement: Supplementary file 1 [file txd-12-e1993-s001.pdf]

Supplementary Table 1. Normal values calculated based on available data on 70 consecutive baseline samples from experiments ranging from June 2024 to April 2026.

Parameter	Mean	SD
pH	7.42	0.05
$PaCO_2$ mmHg	54.8	5.6
PaO_2 mmHg	59.8	12.1
HCO_3^- mmol/L	31.9	2.2
Base excess mmol/L	10.5	3.0
Hemoglobin g/L	93.8	7.7
Sodium mmol/L	137.8	1.4
Potassium mmol/L	3.9	0.2
Calcium mmol/L	1.39	0.09
Chloride mmol/L	101.1	2.1
Glucose mmol/L	4.5	0.8
Lactate mmol/L	1.0	0.4
Creatinine mg/dL	0.96	0.13
BUN mg/dL	6.4	1.7
AST U/L	28.1	6.4

SD: standard deviation; $PaCO_2$: partial pressure of arterial carbon dioxide; PaO_2: partial pressure of arterial oxygen; HCO_3^-: bicarbonate; AST: aspartate aminotransferase; BUN: blood urea nitrogen
